# Employ of Anthocyanins in Nanocarriers for Nano Delivery: In Vitro and In Vivo Experimental Approaches for Chronic Diseases

**DOI:** 10.3390/pharmaceutics14112272

**Published:** 2022-10-24

**Authors:** Ana C. Gonçalves, Amílcar Falcão, Gilberto Alves, João A. Lopes, Luís R. Silva

**Affiliations:** 1 CICS-UBI—Health Sciences Research Centre, University of Beira Interior, 6201-001 Covilhã, Portugal; 2 CIBIT—Coimbra Institute for Biomedical Imaging and Translational Research, University of Coimbra, 3000-548 Coimbra, Portugal; 3 Laboratory of Pharmacology, Faculty of Pharmacy, University of Coimbra, 3000-548 Coimbra, Portugal; 4 iMed.ULisboa, Research Institute for Medicines, Faculdade de Farmácia, University of Lisboa, 1649-003 Lisboa, Portugal; 5 CPIRN-UDI/IPG, Center of Potential and Innovation of Natural Resources, Research Unit for Inland Development (UDI), Polytechnic Institute of Guarda, 6300-559 Guarda, Portugal

**Keywords:** bioactive compounds, anthocyanins, drug delivery system, nanoparticles, chronic diseases, health benefits

## Abstract

Anthocyanins are among the best-known phenolic compounds and possess remarkable biological activities, including antioxidant, anti-inflammatory, anticancer, and antidiabetic effects. Despite their therapeutic benefits, they are not widely used as health-promoting agents due to their instability, low absorption, and, thus, low bioavailability and rapid metabolism in the human body. Recent research suggests that the application of nanotechnology could increase their solubility and/or bioavailability, and thus their biological potential. Therefore, in this review, we have provided, for the first time, a comprehensive overview of in vitro and in vivo studies on nanocarriers used as delivery systems of anthocyanins, and their aglycones, i.e., anthocyanidins alone or combined with conventional drugs in the treatment or management of chronic diseases.

## 1. Introduction

With the increasing prevalence of chronic diseases worldwide and the emerging need to find safer, more effective, cheaper, and lower toxic new therapeutic agents, it is not surprising that phenolic compounds are receiving special attention and importance, given their characteristics (low price, low toxicity, and few, if any, side effects) and the common belief that they can be more effective than synthetic drugs [1,2]. These secondary metabolites are produced by plants to promote their normal cell metabolism and protect them from abiotic (e.g., humidity, light, temperature, moisture, soil, water, pH, and salinity) and biotic factors (e.g., microbial infections) [3]. Their daily consumption by individuals is also promoted and considered a useful strategy to promote health status and prevent numerous diseases, with few or no side effects, unlike conventional drugs [3,4,5]. In fact, phenolics have been used in traditional medicines since ancient times due to their remarkable ability to relieve gastrointestinal complaints, pain, headaches, colds, coughs, and other ailments [6,7]. Therefore, old proverbs, such as “an apple a day keeps the doctor away”, are more relevant than ever.

**Figure 1:**
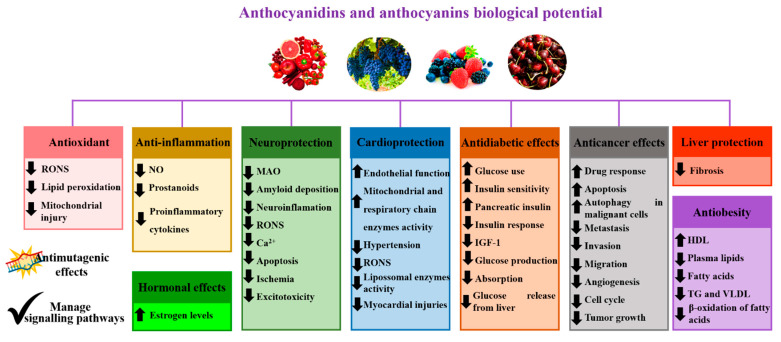
Main biological potential attributed to anthocyanidins and anthocyanins. ↑: increase; ↓: decrease; RONS: reactive oxygen and nitrogen species; NO: nitric oxide; MAO: monoamine oxidase; IGF-1: insulin-like growth factor 1; TG: triglycerides; VLDL: very low-density lipoprotein; HDL: high-density lipoprotein. Adapted from [8,10,11,12,13,14,15,16,17,18,19].

Among these natural molecules, special attention has been paid to anthocyanidins, and their glycosides, i.e., anthocyanins [8]. Both are water-soluble and non-toxic natural compounds, have high molecular weights, and are very affordable. They are considered to be the main contributors to the blue–violet and red colors of many fruits, flowers, and vegetables [9]. In addition, there is a great demand and many potential applications because they show remarkable effects as antioxidant, anti-inflammatory, antimutagenic, antidiabetic, and anticancer agents, both in vitro and in vivo; provide neurological and cardiovascular protection; improve memory (Figure 1); and can be used as additives for beverages, foods, and cosmetics [8,10,11,12,13,14,15,16,17,18,19]. However, anthocyanidins and anthocyanins have low bioavailability because they are poorly absorbed and rapidly metabolized and excreted, which, in turn, compromises their biological and pharmacological benefits [13]. In addition, their high susceptibility to environmental factors, such as chemical factors (e.g., ascorbic acid, light, metal ions, oxygen, pH), processing (e.g., heat), and gastrointestinal digestive processes (enzymes, proteins, and pH), makes them highly sensitive, reactive, unstable, and susceptible to decomposition [9,20]. Additionally, both have the ability to irreversibly bind with some molecules, resulting in structural changes, and, consequently, making them inaccessible for absorption [13].

In view of the above, and in order to overcome these drawbacks and improve their pharmacokinetic, pharmacodynamic, and therapeutic properties, i.e., their bioaccessibility, bioactivity, absorption, stability in the gastrointestinal tract, solubility, and/or better control of their release, the development of novel delivery systems may be an important approach. This is mainly due to their ability to form a physical barrier between anthocyanins and destabilizing factors, protecting them from degradation and enhancing their biological potential [21,22,23,24,25,26]. Several methods exist for the improvement of drug delivery, namely physical (electrospinning/electrospraying, freeze- and spray-drying, lyophilization), chemical (complexation processes), and/or a combination of both (liposomal systems, emulsification, gelation, coacervation) [13,20]. The main biopolymers used for encapsulation are polysaccharides such as cellulose, chitosan, mucilages, natural gums, pectin, starch, and derivatives and proteins such as caseinate, gelatin, soy protein, and whey [9,27,28,29]. Moreover, the encapsulation of anthocyanins also allows for their use at the industrial level [30]. Comparing the nano and microencapsulation, the former has been preferred due to its higher stability and encapsulation efficiency [28]. Therefore, taking into account all these aspects, the main objective of this work was to provide a comprehensive review of in vitro and in vivo studies on nanocarriers employed to deliver anthocyanins and anthocyanidins alone or in combination with active pharmaceutical ingredients.

## 2. Study Selection

In August 2022, a detailed and comprehensive search was conducted on Google Scholar, Medline (PubMed), National Center for Biotechnology Information (NCBI), ResearchGate, Science Direct, Scopus, SpringerLink, and Web of Science databases to find publications on the therapeutic in vitro and in vivo effects of encapsulated anthocyanins and anthocyanidins. The following free terms, keywords, or MeSH terms were used: anthocyanins, anthocyanidins, in vivo, antioxidant potential, anti-inflammatory, chronic disease, encapsulation, drug delivery systems, health-promoting properties, health benefits, nanoparticles, nanocapsules, nanostructures, nanoparticle technology, bioactive compounds, bioactivity, bioavailability and absorption, combined with the operator AND, OR, or NOT. There were no restrictions on the literature search in terms of author(s) or type of publication. All selected articles were written in English. The final database included 211 references, with most of the selected articles used in this review from 2016–2022.

## 3. Anthocyanins: Upcoming Remarkable Active Molecules

**Figure 2:**
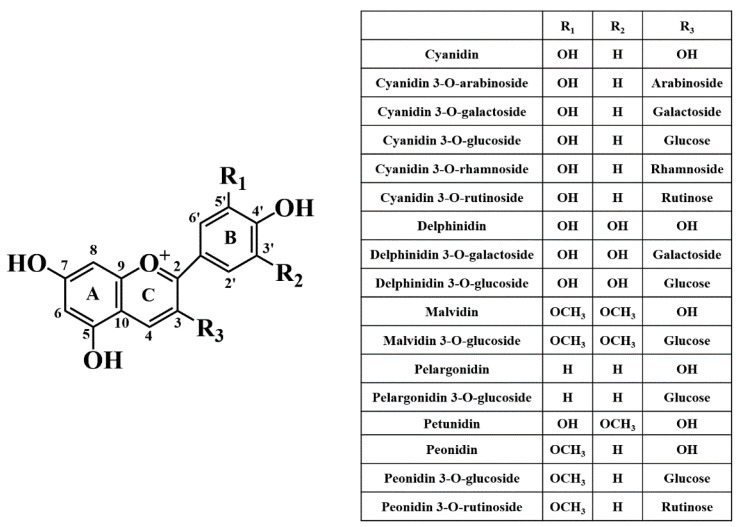
Basic chemical structure of the main anthocyanidins and their glycosides anthocyanins found in nature.

Anthocyanidins and their glycosides (i.e., anthocyanins) are natural pigments synthesized through the general flavonoid pathway, with their main role to protect plants from stress factors and promote normal cellular metabolism [3,31]. To date, about 30 different aglycones have been described [32]. Among them, cyanidin, delphinidin, malvidin, pelargonidin, peonidin, and petunidin are the most abundant (Table 1 and Figure 2) [33]. Like other flavonoid compounds, anthocyanidins and anthocyanins have a carbon skeleton of C6 (A ring)-C3 (C ring)-C6 (B ring), with the C-ring a heterocyclic ring with an oxygen atom (Figure 2) [31]. Once-unstable aglycones are found in nature mainly in conjugation with sugar residues, usually found at carbon 3 [31]; additional sugars are usually attached to carbons 5 and/or 7 or, less commonly, to carbons 3′ and 5′ [34,35]. In general, the predominant sugars are arabinose, galactose, glucose, rhamnose, rutinose, and xylose [36] (Figure 2). These sugar residues can also be acylated with aliphatic acids (e.g., acetic malonic, malic, and oxalic acids) and/or aromatic acids (e.g., caffeic, *ρ*-coumaric, ferulic, sinapic, and *ρ*-hydroxybenzoic acids) [31]. Therefore, it is not surprising that the number and type of sugar units attached to the aglycone, as well as the number, position, and degree of methylation of the hydroxyl groups, and the position of aromatic and/or carboxylated aliphatic acids on the sugar residue affect their biological potential [36,37,38,39]. In general, hydroxylation and methoxylation are considered to be the major contributors to the color and stability of anthocyanins [36,37,38]. Basically, as the number of hydroxyl groups increases, they tend to turn blue but their stability is lower [40]. On the other hand, methoxylation on the B ring mediated by *O*-methyltransferases increases their stability and favors the appearance of red colors [38]. For example, due to its numerous hydroxyl groups, delphinidin shows higher antioxidant activity than petunidin, which, in turn, has the lowest antioxidant activity among the six most abundant anthocyanins [36].

**Figure 3:**
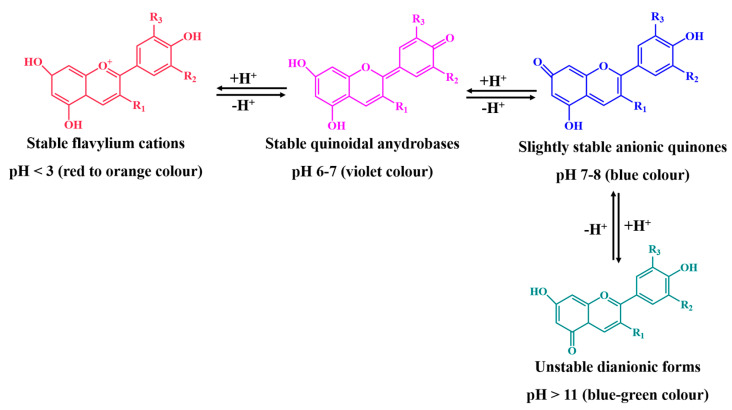
Structural forms and colors shown by anthocyanins at different pH values (R_1_ = H or sugar moiety; R_2_ and R_3_ = hydrogen atom or methyl group).

Moreover, different pH conditions lead to different colors and affect the stability of anthocyanins [41]. As far as we know, anthocyanins are more stable under acidic conditions [31]. Moreover, pH values below 3 contribute to the formation of red and orange anthocyanin compounds, as flavylium cations predominate here, and increase their water solubility, while pH values of 6 to 7 favor the appearance of violet species and pH values above 7 to 8 lead to the appearance of the blue color (Figure 3) [42,43,44]. In contrast, at basic pH, anthocyanins are oxidized and degraded, losing their color as they kinetically and thermodynamically compete for the hydration of flavylium ions, leading to the formation of carbinol pseudobases, chalcones, and quinonoid anions [31,45]. The chemical degradation of chalcones leads to the formation of phenolic acids [9,46].

Other factors that increase the sensitivity of anthocyanins include storage and processing conditions, temperature, concentration, and exposure to light, oxygen, other phenolic compounds, proteins (such as enzymes), molecules (such as water), sulfites, and metal ions [21,40,47,48,49].

### 3.1. Anthocyanin-Rich Sources

Anthocyanins and their aglycones are the main contributors to the color of many fruits, flowers, and vegetables and are considered the key of the biological potential associated with these products [39]. The recommended daily intake is over 100 mg and ranges up to 1000 mg per day. However, Zamora-Ros et al. [50] reported that the daily intake of anthocyanins in Europe ranges from 19.3 to 64.88 mg in men and from 18.73 to 44.08 mg in women. In contrast, in the United States of America (USA), daily consumption ranges from 180–215 mg [51]. Among anthocyanin-rich sources, in Europe, apples, berries, grapes, and pears (50%), and red wine (21%) are the main contributors to daily anthocyanin intake, while in the USA, berries, wine, grapes, and bananas (20, 16, 11, and 11%, respectively) are the main sources [52].

Indeed, anthocyanins possess remarkable and recognized health-promoting properties, so, it is highly recommended to increase their consumption to promote health status and prevent the occurrence and/or development of chronic diseases [19,29,53]. Thus, strong correlations have already been reported between the total amount of anthocyanins extracted from sweet cherries and the ability to inhibit the enzyme *α*-glucosidase (r = 0.9929) and to affect the growth of colon adenocarcinoma Caco-2 cells (r = 0.6674) [11]. In addition, recent studies have shown that anthocyanins have a remarkable ability to reduce oxidative stress in hepatocellular HepG2 carcinoma cells [10], as well as impair the growth of AGS gastric cancer cells and reduce inflammatory iNOs and COX-2 expression and resulting nitric oxide radicals on RAW 264.7 macrophages [12].

Anthocyanidins and anthocyanins are widely distributed in nature [39]. The most important of these are listed in Table 1. Cyanidin derivatives are the most abundant in plants, especially cyanidin 3-*O*-glucoside and 3-*O*-rutinoside, followed by delphinidin and pelargonidin glycosides, and finally peonidin, malvidin, and petunidin methylated derivatives [54]. As for fruits, açaí is probably one of the most anthocyanin-rich fruits, with a total anthocyanin content (TAC) varying between 1020.00–1433.00 mg of cyanidin 3-*O*-glucoside (C3G) per 100 g fresh weight (fw), with a predominance of cyanidin 3-*O*-rutinoside and cyanidin 3-*O*-glucoside [55,56]. Red berries also have considerable amounts of anthocyanins and are capable of providing substantial amounts of anthocyanin doses in a single serving, contrasting with blueberries, whose TAC values range from 34.50 to 552.20 and from 69.97 to 378.31 mg C3G per 100 g of fw for highbush and rabbit-eye blueberries, respectively [57,58,59,60,61,62]. Regarding the profile of anthocyanins in highbush blueberries, they have higher amounts of malvidin 3-*O*-galactoside, peonidin 3-*O*-glucoside, delphinidin 3-*O*-glucoside, and delphinidin 3-*O*-galactoside, while rabbit-eye blueberries are richer in peonidin 3-*O*-glucoside, malvidin 3-*O*-glucoside, malvidin 3-*O*-arabinoside, and delphinidin 3-*O*-galactoside [57,63]. Elderberries and chokeberry also have considerable amounts of anthocyanins (130.80–953.00 and 428.00 mg C3G per 100 g of fw, respectively), as do blueberries (280.98–329.00 mg C3G per 100 g of fw), haskap (88.30–273.00 mg C3G per 100 g of fw), black currants (62.80–271.33 mg C3G per 100 g of fw), red grapes (0.30–164.20 mg C3G per 100 g of fw), and red raspberries (21.90–62.50 mg C3G per 100 g of fw) [64,65,66,67,68,69,70]. In addition, sour and sweet cherries also have significant amounts of anthocyanins, with TAC values ranging from 10.50–87.40 mg C3G per 100 g of fw for tart cherries and 1.03–179.14 mg C3R per 100 g of dw for sweet cherries [71,72,73]. Comparing the anthocyanin profile of both cultivars, sweet cherries have higher levels of cyanidin 3-*O*-rutinoside and cyanidin 3-*O*-glucose, while cyanidin 3-*O*-glucosyl-rutinoside is the predominant anthocyanin in sour cherries [72,74,75,76,77].

In vegetables, TAC levels around 150 mg C3G per 100 g fw are found in eggplant, purple sweet potato, and red cabbage. Among anthocyanins, delphinidin 3-*O*-rutinoside is the predominant one in eggplant [78], while 3-*O*-sophoroside-5-glucoside derivatives of peonidin and cyanidin are the most important in purple sweet potato [79]. On the other hand, cyanidin-3-diglucoside-5-glucoside, cyanidin-3-(sinapoyl)(sinapoyl)-diglucoside-5-glucoside, and cyanidin-3-(*ρ*-coumaroyl)-diglucoside-5-glucoside are most commonly found in red cabbage [80].

Compare to beverages, elderberry juices are richest in anthocyanins, with values ranging from 90.00 to 5270.00 mg C3G per liter [81]. Among anthocyanins, cyanidin-3-*O*-sambubioside predominates, followed by cyanidin-3-*O*-glucoside and cyanidin-3-*O*-sambubioside-5-*O*-glucoside [82]. In addition, blueberry juices also contain considerable amounts of anthocyanins, with values ranging from 317.07 to 588.87 mg C3G per liter [83]. In contrast to elderberry juices, malvidin 3-*O*-glucoside and malvidin 3-*O*-galactoside are most abundant in blueberry juices [84]. Raspberry juices are also rich in anthocyanins, having TAC values around 336.70 mg C3G per liter [85]. In addition, pomegranate juices and red wines exhibit similar amounts of anthocyanins (with average values around 200 mg C3G per liter) [85,86,87,88].

Of course, it should be noted that anthocyanin content is highly dependent on genotype/variety, climate, ripening stage, local origin, and agronomic and postharvest practices and conditions [89]. For example, Howard et al. [90] reported that blueberry jams stored at 4 °C had 65% more anthocyanins after 6 months of storage than those stored at 25 °C. Similar data were obtained for their juices after an 8-month study [84].

**Table 1:** Main anthocyanin-rich natural sources.

Main Sources	Scientific Name	Total Anthocyanins Content (mg C3G/100 g fw)	Main Anthocyanins Found	References
*Fruits*				
Açaí	*Euterpe oleracea*	1020.00–1433.00	Cyanidin 3-(acetyl)hexose Cyanidin 3-*O*-glucoside Cyanidin 3-*O*-rutinoside Cyanidin 3,5-hexose pentose Pelargonidin 3-*O*-glucoside Peonidin 3-*O*-glucoside Peonidin 3-*O*-rutinoside	[55,56]
Acerola	*Malphigia emarginata*	23.00–48.00	Cyanidin 3-*O*-rhamnoside Pelargonidin 3-*O*-rhamnoside Cyanidin Pelargonidin	[56,91]
Apple	*Malus domestica*	<0.01–8.22	Cyanidin 3-*O*-diglucoside-5-*O*-glucoside Cyanidin 3-*O*-(6″-*ρ*-coumaroyl-glucoside) Peonidin 3-*O*-sambubioside-5-*O*-glucoside Cyanidin 3,5-*O*-diglucoside Delphinidin 3-*O*-xyloside Delphinidin 3-*O*-glucosyl-glucoside Cyanidin 3-*O*-(2-*O*-(6-*O*-(E)-caffeoyl-D glucoside)-D-glucoside)-5-*O*-D-glucoside Delphinidin 3-*O*-galactoside	[92,93]
Bilberry	*Vaccinium myrtillus*	280.98–329.00	Cyanidin 3-*O*-arabinoside Cyanidin 3-*O*-galactoside Cyanidin 3-*O*-glucoside Delphinidin 3-*O*-arabinoside Delphinidin 3-*O*-galactoside Delphinidin 3-*O*-glucoside Malvidin 3-*O*-arabinoside Malvidin 3-*O*-galactoside Malvidin 3-*O*-glucoside Petunidin 3-*O*-arabinoside Petunidin 3-*O*-galactoside Petunidin 3-*O*-glucoside Peonidin 3-*O*-galactoside Peonidin 3-*O*-glucoside	[67,94,95]
Blackberry	*Rubus fruticosus*	12.30–150.00	Cyanidin 3-*O*-glucoside Cyanidin 3-*O*-rutinoside Cyanidin 3-*O*-xyloside Cyanidin 3-*O*-malonyl-glucoside Cyanidin 3-dioxalylglucoside	[96,97]
Black currant	*Ribes rubrum*	62.80–271.33	Cyanidin 3-*O*-glucoside Cyanidin 3-*O*-rutinoside Delphinidin 3-*O*-glucoside Delphinidin 3-*O*-rutinoside	[92,98]
Rabbiteye blueberry	*Vaccinium ashei*	69.97–378.31	Cyanidin 3-*O*-arabinoside Cyanidin 3-*O*-galactoside Cyanidin 3-*O*-glucoside Delphinidin 3-*O*-arabinoside Delphinidin 3-*O*-galactoside Delphinidin 3-*O*-glucoside Malvidin 3-*O*-arabinoside Peonidin 3-*O*-glucoside Peonidin 3-*O*-arabinoside Peonidin 3-*O*-galactoside Petunidin 3-*O*-galactoside Petunidin 3-*O*-glucoside Petunidin 3-*O*-arabinoside	[57,58,59,60]
Highbush blueberry	*Vaccinium corymbosum*	34.50–552.20	Cyanidin 3-*O*-arabinoside Cyanidin 3-*O*-galactoside Cyanidin 3-*O*-glucoside Delphinidin 3-*O*-galactoside Delphinidin 3-*O*-glucoside Delphinidin 3-*O*-arabinoside Malvidin 3-*O*-arabinoside Malvidin 3-*O*-galactoside Malvidin 3-*O*-glucoside Petunidin 3-*O*-galactoside Petunidin 3-*O*-glucoside Peonidin 3-*O*-arabinoside Peonidin 3-*O*-galactoside Petunidin 3-*O*-arabinoside	[58,60,61,62]
Cranberry	*Vaccinium macrocarpon*	11.10–32.00	Cyanidin 3-*O*-galactoside Cyanidin 3-*O*-glucoside Cyanidin 3-*O*-xyloside	[65,99]
Chokeberry	*Aronia melanocarpa*	428.00	Cyanidin 3-*O*-arabinoside Cyanidin 3-*O*-galactoside Cyanidin 3-*O*-glucoside Cyanidin 3-*O*-xyloside	[65]
Elderberry	*Sambucus nigra*	130.80–953.00	Cyanidin 3,5-diglucoside Cyanidin 3-sambubioside-5-glucoside Cyanidin-3-*O*-glucoside Cyanidin 3-*O*-sambubioside	[64,67,100,101]
Fig	*Ficus carica*	2.57–9.70	Cyanidin 3,5-diglucoside Cyanidin 3-*O*-glucoside Cyanidin 3-*O*-rutinoside	[102,103]
Lingonberry	*Vaccinium vitis-idaea*	45.00	Cyanidin 3-*O*-arabinoside Cyanidin 3-*O*-galactoside Cyanidin 3-*O*-glucoside Peonidin3-*O*-glucoside	[65]
Haskap	*Lonicera caerulea*	88.30–273.00	Cyanidin 3,5-diglucoside Cyanidin 3-*O*-glucoside Cyanidin 3-*O*-rutinoside Pelargonidin 3-*O*-glucoside Peonidin 3-*O*-glucoside Peonidin 3,5-diglucosid	[104,105,106]
Nectarine	*Prunus persica* var. *nucipersica*	0.21–0.59	Cyanidin 3-*O*-glucoside Cyanidin 3-*O*-rutinoside	[107,108]
Peach	*Prunus persica*	0.15–0.44	Cyanidin-3-*O*-glucoside, Cyanidin-3-*O*-rutinoside	[109,110]
Plum	*Prunus domestica*	13.70–33.11	Cyanidin 3-*O*-glucoside Cyanidin 3-*O*-rutinoside Cyanidin 3-*O*-xyloside Peonidin 3-*O*-glucoside Peonidin 3-*O*-rutinoside	[92,111]
Pomegranate	*Punica granatum*	17.95–36.41 ^1^	Cyanidin-3,5- diglucoside Cyanidin 3-*O*-glucoside Delphinidin 3-*O*-glucoside Delphinidin-3,5- diglucoside Pelargonidin 3-*O*-glucoside Pelargonidin-3,5- diglucoside	[112,113,114]
Red currant	*Ribes rubrum*	7.10–19.30	Cyanidin 3-*O*-glucoside Cyanidin 3-*O*-rutinoside Delphinidin Delphinidin 3-*O*-glucoside Delphinidin 3-*O*-rutinoside	[115,116]
Red grape	*Vitis vinifera*	0.30–164.20	Delphinidin 3-*O*-glucoside Cyanidin 3-*O*-glucoside Petunidin 3-*O*-glucoside Peonidin 3-*O*-glucoside Malvidin 3-*O*-glucoside Cyanidin 3-(acetyl)hexose Petunidin 3-(acetyl)glucoside Peonidin 3-(acetyl)glucoside Malvidin 3-(acetyl)glucoside Peonidin 3-*ρ*-coumaroyl glucoside Malvidin 3-*ρ*-coumaroyl glucoside	[66,117,118,119]
Red pear	*Pyrus communis*	1.20–12.0 ^1^	Cyanidin 3-*O*-galactoside Cyanidin 3-*O*-glucoside Cyanidin pentoside Cyanidin 3-*O*-arabinoside Cyanidin 3-*O*-rutinoside Peonidin 3-*O*-galactoside Peonidin 3-*O*-galactoside	[120,121]
Red raspberry	*Rubus idaeus*	21.90–62.50	Cyanidin 3-*O*-sophoroside Cyanidin 3-(2-glucosylrutinoside) Cyanidin 3-*O*-glucoside Cyanidin 3-(2-glucosylrutinoside) Cyanidin 3-*O*-xylosylrutinoside Pelargonidin 3-*O*-sophoroside Cyanidin 3-*O*-rutinoside Pelargonidin 3-(2-glucosylrutinoside) Pelargonidin 3-*O*-glucoside Pelargonidin 3-*O*-rutinoside	[69,70]
Strawberry	*Fragaria × ananassa*	29.00–49.43	Cyanidin 3-*O*-glucoside Cyanidin 3-*O*-rutinoside Cyanidin 3-malonylglucose-5-glucose Delphinidin 3-*O*-glucoside Malvidin 3-*O*-glucoside Pelargonidin 3-*O*-glucoside Pelargonidin 3-*O*-rutinoside Pelargonidin 3-(acetyl)glucoside	[67,92,122,123]
Sweet cherry	*Prunus avium*	1.03–179.14 *	Cyanidin 3-*O*-glucoside Cyanidin 3-*O*-rutinoside Delphinidin 3-*O*-rutinoside Peonidin 3-*O*-rutinoside	[71,74,75]
Tamarillo	*Solanum betaceum*	29.70–481.37 ^2^	Cyanidin 3-*O*-glucoside Cyanidin 3-*O*-rutinoside Delphinidin 3-*O*-rutinoside Pelargonidin 3-*O*-rutinoside	[124]
Sour cherry	*Prunus cerasus*	10.50–87.40	Cyanidin 3-*O*-glucorutinoside Cyanidin-3-O-glucoside Cyanidin-3-O-rutinoside	[73,125]
Tomato	*Solanum lycopersicum*	4.35–7.09 ^1^	Delphinidin 3-(cis-*ρ*-coumaroyl)-rutinoside-5-glucoside Petunidin 3-(caffeoyl)-rutinoside-5-glucoside Delphinidin 3-(*trans*-*ρ*-coumaroyl)-rutinoside-5-glucoside Delphinidin 3-(feruloyl)-rutinoside-5-glucoside Petunidin 3-(*cis*-*p*-coumaroyl)-rutinoside-5-glucoside Petunidin 3-(*trans*-*ρ*-coumaroyl)-rutinoside-5-glucoside Petunidin 3-(feruloyl)-rutinoside-5-glucoside Malvidin 3-(*cis*-*ρ*-coumaroyl)-rutinoside-5-glucoside Malvidin 3-(*trans*-*ρ*-coumaroyl)-rutinoside-5-glucoside Malvidin 3-(feruloyl)-rutinoside-5-glucoside Petunidin 3-(*trans*-*ρ*-coumaroyl-rhamonside)-glucoside-5-glucoside Malvidin 3-(*ρ*-methoxy-*trans*-coumaroyl)-rutinoside-5-glucoside	[126,127]
*Vegetables*				
Black carrot	*Daucus carota* ssp. *sativus* var. *atrorubens*	1.50–17.70	Cyanidin 3-xylosyl(glucosyl)galactoside Cyanidin 3-xylosylgalactoside Cyanidin 3-xylosyl(*ρ*-hydroxybenzoylglucosyl) galactoside Cyanidin 3-xylosyl(sinapoylglucosyl)galactoside Cyanidin 3-xylosyl(feruloylglucosyl)galactoside Cyanidin 3-xylosyl(coumaroylglucosyl)galactoside Pelargonidin 3-xylosyl(feruloylglucosyl)galactoside Peonidin 3-xylosylgalactoside Peonidin 3-xylosyl(sinapoylglucosyl)galactoside Peonidin 3-xylosyl(feruloylglucosyl)galactoside	[128]
Eggplant	*Solanum melongena*	14.86–182.44	Delphinidin 3-rutinoside-5-glucoside Delphinidin 3-rutinoside-glucoside Delphinidin 3-*O*-glucoside Delphinidin 3-*O*-rutinoside	[78,129]
Purple sweet potato	*Dioscorea alata*	4.91–150.00	Cyanidin 3-sophoroside-5-glucoside Peonidin 3-sophoroside-5-glucoside Cyanidin 3-*ρ*-hydroxybenzoylsophoroside-5-glucoside Peonidin 3-*ρ*-hydroxybenzoylsophoroside-5-glucoside Delphinidin 3,5-diglucoside Cyanidin 3-(6″-feruloylsophoroside)-5-glucoside Peonidin 3-(6″-feruloylsophoroside)-5-glucoside Cyanidin 3-(6″-caffeoylsophoroside)-5-glucoside Cyanidin 3-caffeoyl-*ρ*-hydroxybenzoyl sophoroside-5-glucoside Cyanidin 3-(6″-caffeoyl-6″-feruloylsophoroside)-5-glucoside Peonidin 3-caffeoyl sophoroside-5-glucoside Peonidin-3-caffeoyl-*ρ*-hydroxybenzoyl sophoroside-5-glucoside Peonidin-3-(6″-caffeoyl-6″-feruloylsophoroside)-5-glucoside	[79,130,131]
Red cabbage	*Brassica oleracea* var. *capitata f. rubra*	109.00–188.00	Cyanidin 3-*O*-diglucoside-5-*O*-glucoside Cyanidin 3-(*ρ*-coumaroyl)-*O*-diglucoside-5-*O*-glucoside Cyanidin 3-(feruloyl)-*O*-diglucoside-5-*O*-glucoside Cyanidin 3-(sinapoyl)-*O*-diglucoside-5-*O*-glucoside Cyanidin 3-(feruloyl) (feruloyl)-*O*-diglucoside-5-*O*-glucoside Cyanidin 3-(feruloyl) (sinapoyl)-*O*-diglucoside-5-*O*-glucoside Cyanidin 3-(sinapoyl) (sinapoyl)-*O*-diglucoside-5-*O*-glucoside	[80,132,133]
Red chicory	*Cichorium intybus*	43.68–72.77	Cyanidin 3-*O*-glucoside Cyanidin 3-*O*-rutinoside Cyanidin 3-*O*-(6″malonyl)-glucoside Pelargonidin 3-*O*-glucoside	[134,135]
Red onion	*Allium cepa*	29.99	Cyanidin 3-*O*-glucoside Cyanidin 3-*O*-laminaribioside Cyanidin 3-*O*-(3″-malonylglucoside) Peonidin 3-*O*-glucoside Delphinidin 3,5-*O*-diglucoside Cyanidin 3-*O*-(6″-malonylglucoside) Cyanidin 3-*O*-(6″-malonyl-laminaribioside) Peonidin 3-*O*-(6″-malonylglucoside) Delphinidin	[136,137]
*Beverages*				
Blackberry juice	*Rubus fruticosus*	12.30–107.00	Cyanidin 3-*O*-glucoside Cyanidin 3-*O*-rutinoside Cyanidin 3-*O*-xyloside Cyanidin 3-*O*-malonyl-glucoside Cyanidin 3-dioxalylglucoside	[96]
Blueberry juice	*Vaccinium ashei*	317.07–588.87 ^3^	Delphinidin 3-*O*-galactoside Delphinidin 3-*O*-glucoside Cyanidin 3-*O*-galactoside Delphinidin 3-*O*-arabinoside Cyanidin 3-*O*-glucoside Petunidin 3-*O*-galactoside Cyanidin 3-*O*-arabinoside Petunidin 3-*O*-glucoside Peonidin 3-*O*-galactoside Petunidin 3-*O*-arabinoside Malvidin 3-*O*-galactoside Malvidin 3-*O*-glucoside Peonidin 3-*O*-arabinoside Malvidin 3-*O*-arabinoside Delphinidin 3-*O*-(6″-acetylglucoside) Petunidin 3-*O*-(6″-acetylglucoside) Malvidin 3-*O*-(6″-acetylglucoside)	[83,84]
Elderberry juice	*Sambucus nigra*	90.00–5270.00 ^3^	Cyanidin 3-*O*-sambubioside-5-*O*-glucoside Cyanidin 3-*O*-sambubioside Cyanidin 3-*O*-glucoside	[81,82,85]
Pomegranate juice	*Punica granatum*	187.00–291.00 ^3^	Cyanidin 3-*O*-glucoside Cyanidin 3,5-diglucoside Cyanidin 3-*O*-pentose Cyanidin pentose-hexose Delphinidin 3-*O*-pentose Delphinidin 3,5-diglucoside Pelargonidin 3-*O*-glucoside Pelargonidin 3,5-diglucoside	[86,87,138]
Raspberry juice	*Rubus idaeus*	336.70 ^3^	Cyanidin 3-*O*-glucoside Cyanidin 3-*O*-sophoroside Cyanidin 3-*O*-rutinoside Cyanidin 3-*O*-glucorutinoside Pelargonidin 3-*O*-sophoroside Pelargonidin 3-rutinoside-3′-glucoside	[85,139,140]
Red wine	*Vitis vinifera*	52.61–201.60 ^3^	Cyanidin 3-*O*-glucoside Delphinidin 3-*O*-glucoside Malvidin 3-(acetyl)glucoside Malvidin 3-O-coumarylglucoside Malvidin 3-O-glucoside Peonidin 3-*O*-glucoside Peonidin 3-(acetyl)glucoside Peonidin 3-*O*-coumarylglucoside Petunidin 3-*O*-glucoside	[85,88,141]
Strawberry juice	*Fragaria × ananassa*	63.60 ^3^	Cyanidin 3-*O*-glucoside Pelargonidin-3-*O*-rutinoside Pelargonidin 3-*O*-glucoside	[85,140]
Tart cherry juice	*Prunus cerasus*	350.00–633.50 ^3^	Cyanidin Cyanidin 3-*O*-sophoroside Cyanidin 3-*O*-glucosylrutinoside Cyanidin 3-*O*-glucoside Cyanidin 3-*O*-rutinoside Peonidin 3-*O*-rutinoside	[47,77,142]

C3G: cyanidin 3-*O*-glucoside; fw: fresh weight; * mg cyanidin 3-*O*-rutinoside (C3R)/100 g of fw; ^1^ identified by HPLC technique; ^2^ identified by HPLC technique (mg/100 g dried weight); ^3^ mg C3G/L.

### 3.2. Absorption, Distribution, Metabolism, and Excretion (ADME) and Bioavailability of Anthocyanins

Although there are existing studies on the ADME processes and the bioavailability of anthocyanins, understanding these issues is still a major challenge [143]. However, they are necessary to fully reveal and explore the biological potential of anthocyanins [9,41]. Nowadays, it is believed that the absorption of anthocyanins is higher than the expectable (<28.00%) once anthocyanins undergo extensive metabolism in the colon [33,144,145,146]. Nevertheless, their percentages are highly variable, largely depending on the individual’s sex, genetic profile, age, pre-systemic metabolism, physiological and pathological conditions, dietary habits, lifestyle, intestinal flora, and the presence of possible intolerances, as well as chemical structure, molecular size, glycosylation pattern, and behavior in the gastrointestinal tract, including solubility, transport, and permeability [9,147]. For example, pelargonidin aglycone and derivatives are more readily absorbed than other anthocyanins when they have lower substituents on the B ring [148]. Comparing the sugar residues, malvidin 3-*O*-arabinoside shows higher absorption than its 3-*O*-glucoside derivative [149]. In addition, it is important to consider the maturity degree of the food matrix, while cooking practices can also influence the availability process, as well as their levels and ability to be released from the food matrix, and whether they are administered alone or with other foods and beverages, and/or after a fasting period [41]. Nevertheless, anthocyanins are generally readily absorbed, reaching maximum concentrations ranging from 1.4 to 592 nmol/L (doses of 68–1300 mg) 30 min to 4 h after ingestion [150,151]. The recovery rate in urine is less than 2% 24 h after ingestion [152].

As mentioned earlier, most anthocyanins found in foods are glycosides [9]. After consumption, anthocyanins are partially degraded by the microbiota in the mouth [153]. They are then passed to the stomach, where the low pH promotes the stability of anthocyanins and their persistence in the form of glycosides [22]. Unlike other flavonoids, anthocyanins can be absorbed along with their sugar moieties [143]. They require the action of bilitranslocase because they are unable to cross cell membranes by passive diffusion due to their hydrophilic nature [49,148]. Therefore, anthocyanins are delivered by bilitranslocase to the portal vein of the liver to be distributed by systemic circulation to target tissues and organs (bioavailability) [148].

However, the majority of anthocyanins ingested in the diet are transported by glucose transporters (GLUT) 1 and 3 to the small intestine, where they are degraded, possibly increasing their absorption and thus bioavailability [41,49]. For this purpose, anthocyanins are deglycosylated in a first step by cytosolic *β*-glucosidase (CGB) in the intestinal lumen and lactase-phlorizin hydrolase (LPH) in the brush border of intestinal epithelial cells, producing more lipophilic aglycones that are more easily absorbed [144]. Therefore, the released aglycone can enter the intestinal epithelial cells by passive diffusion or via sodium-dependent transporters GLUT 1 and GLUT 2, metabolic detoxification through phase I (the reactions of hydrolysis, oxidation, and reduction), and phase II enzymatic metabolism (conjugation reactions such as methylation, glucuronidation, and sulfation mediated by the enzyme catechol-*O*-methyltransferase, uridine-5′-diphospho-glucuronosyltransferase, and sulfotransferase mediated, respectively) [41,143,154]. Similar reactions may also occur in the liver and kidneys [154]. The absorption of the non-acylated anthocyanins is four times higher than that of the acylated ones [155].

The non-absorbed anthocyanins can be excreted in the urine and bile [156] or enter the colon, where they are metabolized by 300–500 different bacteria, mainly of the genera *Bacteroides, Bifidobacterium, Clostridium*, and *Eubacterium*, which convert them to lower molecular-weight compounds, namely phenolic acids, aldehydes, or other phenolics, to increase their absorption [41,144,153]. For example, it has been previously reported that 16 different metabolites derived from the degradation of anthocyanins were identified after the consumption of red raspberries [154]. In addition, 29 different metabolites were detected in the urine of 20 volunteers who had consumed anthocyanin-containing beverages including caffeic, *trans*-isoferulic, vanillic, *trans*-ferulic, hippuric, 2,4,6-trihydroxybenzaldehyd, and 4-dihidroxifenilacetic acids derivatives. They peaked 3.5 h after consumption of the beverage [157]. Moreover, this modulation between colonic bacteria and anthocyanins is very interesting and beneficial because it produces short-chain fatty acids, which together with the new phenolic acids formed, create favorable conditions for the proliferation of probiotic bacteria with remarkable health-promoting properties, such as *Bifidobacteria, Actinobacteria*, and *Lactobacilli* [158]. Thus, the conjugated forms may be excreted through bile to jejunum to be then recycled by enteropathic circulation [156]. Unabsorbed anthocyanins are excreted in the stool [33].

Given the great potential of anthocyanins as dietary supplements, several efforts have been made to increase the stability of anthocyanins in the gastrointestinal tract, their absorption and bioavailability, and thus their biological potential [9]. One of the most promising strategies is the encapsulation of anthocyanins, which will be discussed below [9,48].

## 4. Anthocyanins in Nano-Delivery Systems

Given the toxicity of many synthetic drugs, the search for and development of new effective drugs is essential [4]. Anthocyanins are a target of many studies in this regard [2,159,160,161,162,163].

However, these phytochemicals are very sensitive to external factors (e.g., pH variations, water, temperature) and have a short half-life [164]. Therefore, it is imperative to develop new delivery systems that do not exhibit toxicity and are able to increase the stability of anthocyanins and make them kinetically and thermodynamically stable, as well as improve their solubility and pharmaceutical properties [165].

Among the various alternatives studied, those related to nanotechnology and nanoencapsulation have attracted the greatest interest in many fields, such as medicine, namely in the prevention, diagnosis, and treatment of numerous diseases through active or passive targeting [166,167,168,169,170]. They are preferred over micro-delivery systems because the latter are more unstable in the physiological environment due to their large particle size, low zeta potential, and low encapsulation efficiency [171]. Therefore, various approaches to nanoencapsulation are currently being investigated for the different routes of administration, including intravenous, nasal, oral, parenteral, intraocular, and dermal topical applications [172]. In addition, most of these approaches can also easily cross the blood–brain barrier, which increases the therapeutic potential of the molecule(s) [170,173]. This technique can be applied because anthocyanins are able to generate van der Waals and hydrogen bonds as well as hydrophobic interactions with these nanocarriers, which increases their stability [24,26,165]. The latter has a particle size between 1 and 1000 nm and may consist of organic components (e.g., polymer and lipid-based nanoparticles, such as nanoemulsions, liposomes, and nanoparticles of solid lipid), inorganic coatings (e.g., metallic nanostructures of gold and titanium dioxide, such as nano-quantum dots and nanodiamonds), or a combination of both. Most of them are biodegradable and non-toxic [171,174].

Biopolymers are the most commonly used for nanoencapsulation because they incorporate various types of hydrophobic and hydrophilic molecules (e.g., drugs, proteins, phytochemicals, and other molecules), minimizing their undesirable effects and enhancing their benefits [175,176]. Among them, carbohydrates derived from alginate and chitosan, natural gums, and protein derivatives are the most popular [164,177]. They are very effective in encapsulating anthocyanins and contribute greatly to shifting the flavylium cation structure, protecting them from negative environmental effects, thus promoting their stability in the gastrointestinal tract and increasing their bioavailability [20,160,178]. Thus, several studies have already shown that encapsulation of anthocyanins with chitosan and derivatives can improve their physical and oxidative stability, preserve their antioxidant activity, and promote a slower degradation during simulated gastrointestinal digestion and environmental storage [22,27,164,179,180]. Pectin is also considered a promising coating because of its distinct ability to protect anthocyanins from different pH ranges and temperatures, as well as from damage by ascorbic acid [23,181]. In addition, it promotes a slower release of anthocyanins [182]. Zhao et al. [183] developed a nanoliposome of anthocyanins from blueberries coated with pectin and demonstrated a slower release of anthocyanins in simulated gastric fluid (≤35.9%), but faster release in simulated intestinal fluid, caused by the degradation of vesicles by the enzyme pancreatin. Liposomal micelles were also found to be effective in increasing the stability and resistance of blueberry anthocyanins in the gastrointestinal tract, resulting in a bioavailability greater than 90% [184]. In addition, the combination of pectin and chitosan has also been a subject of numerous studies. So far, they have already shown a promising ability to protect anthocyanins from degradation by milk and white fluorescent light, as well as from different pH and temperature levels, to maintain their antioxidant capacity and to improve retention during simulated ingestion and cellular uptake into human Caco-2 intestinal cells [13,28,185]. Similar results were obtained with gum arabic [186,187], *α*-lactalbumin [48], *β*-lactoglobulin [2], *β*-glucan [21], inulin and oligofructose carbohydrates [5], chondroitin sulfate [24], and casein conjugated with carboxymethyl cellulose [160]. The combination of whey protein with glucose also showed a remarkable ability to protect anthocyanins from high temperatures (80 °C) at acidic pH [188], while the use of ovalbumin conjugated with dextran appears to provide added value for the protection of anthocyanins from hydrogen peroxide-induced oxidative damage, as its conjugation showed a remarkable ability to increase anthocyanins’ chemical stability, cellular uptake, and intestinal absorption in human Caco-2 intestinal cells [26]. Peptides are also promising materials for nanoencapsulation. For example, C6M1, a peptide composed of 18 amino acids, was previously reported to maintain the free radical scavenging ability of cyanidin 3-*O*-glucoside and to be resistant to higher pH values, temperature, and concentrations of metal ions [162]. Apoferritin nanocages exhibit similar capabilities and also show the ability to facilitate the transport of this anthocyanidin through the cell monolayer of Caco-2 cells [189]. Finally, the use of EUDRAGIT^®^ L100 and polyethylene glycol 2000 can also improve the bioavailability of anthocyanins from açaí berries [190].

Nanoencapsulation involves two primary structures: the first is the nanospheres core structure, in which the polymer matrix is dispersed and/or adsorbed by the bioactive compounds, and the second is the nanocapsule, which may consist of water or oil and a polymer shell [171,191]. When nanoparticles reach the outer cell membrane, they can interact with components of the plasma or extracellular membrane and enter the cell by endocytosis [26,192,193]. In addition, some biopolymers allow controlled release of the molecules that they coated at specific times and cells/organs [9,194].

**Figure 4:**
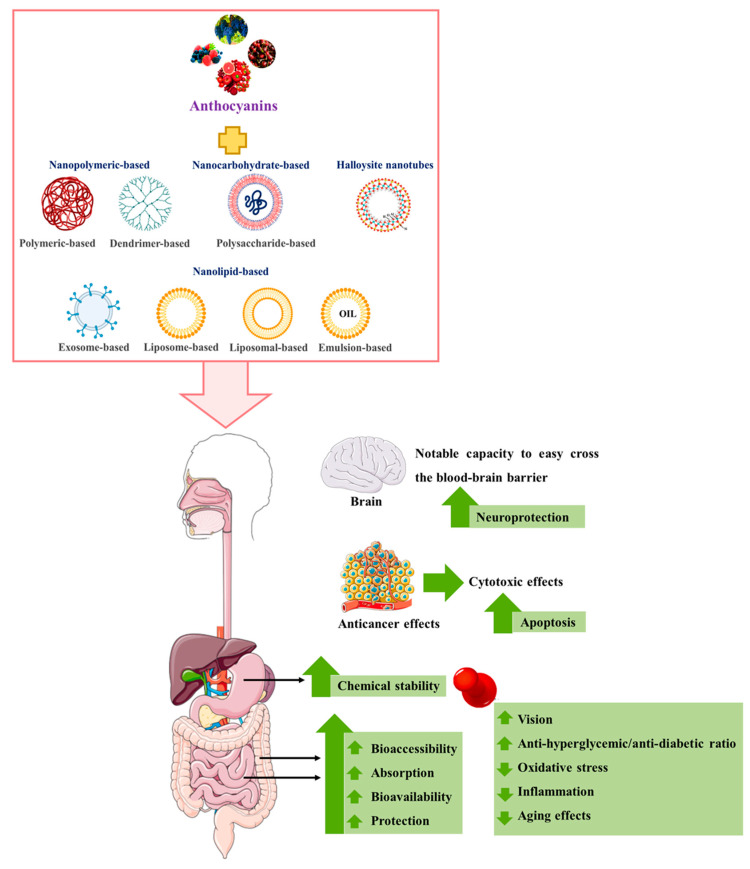
Main in vitro and in vivo effects of anthocyanins-loaded nanocarriers. ↑: increase; ↓: decrease. (Portions of Figure 4 were drawn using images from Servier Medical Art. Servier Medical Art by Servier is licensed under a Creative Commons Attribution 3.0 Unported License (https://creativecommons.org/licenses/by/3.0/) and BioRender.com (https://biorender.com/) (both accessed on 6 August 2022) and from Guimarães [195]).

The most promising in vitro and in vivo studies on the effects of nanoencapsulated anthocyanins on human health are summarized and discussed below (Figure 4 and Table 2).

### 4.1. In Vitro Studies

#### 4.1.1. Nano-Polymeric Coatings

The nanoencapsulation of anthocyanins with polymeric coatings has been commonly performed and investigated in in vitro conditions [193,196,197]. Among the various options, the use of the synthetic polymer poly(lactic-co-glycolic acid)-acid is very popular because it has tremendous potential to form stable complexes, is non-toxic and biodegradable, and the associated results are highly reproducible worldwide [198]. In addition, they display good tissue penetration and are easily manipulated [193]. As far as we know, this synthetic polymer showed great efficiency in coating pelargonidin, at concentrations of 10, 20, and 30 µM, increasing its ability to inhibit the production of reactive oxygen species in L6 muscle cells exposed to the pesticide cypermethrin after 12 and 24 h of exposure [197], as well as protecting these cells from nuclear damage by activating poly(ADP-ribose) polymerase and the p53 pathway, at a concentration of 9 µg/mL [198]. In addition, this coating can also enhance the ability of pelargonidin, at a concentration of 9 µM, to reduce the expression of GLUT 4, insulin receptor substrate 1, peptidase inhibitor 3, glycerol kinase, and protein kinase, and to induce cytochrome complex-mediated apoptosis in alloxan-induced hyperglycemic L6 skeletal muscle cells [196]. Moreover, anthocyanins encapsulated with this polymer also showed a higher capacity to protect human neuroblastoma cells SH-SY5Y from neurodegeneration against A*β*_1–42_-induced toxicity than those not encapsulated. Indeed, encapsulated anthocyanins at concentrations between 50 and 200 µM can remarkably reduce Alzheimer’s disease markers amyloid precursor protein, beta-site amyloid precursor protein cleaving enzyme, neuroinflammatory markers phospho-nuclear factor kappa B, tumor necrosis factor alpha, and inducible nitric oxide synthase after 12 h of exposure, and the neuroapoptotic markers B-cell lymphoma 2 and associated X-protein (Bax) and caspase-3 protein expressions, and showed notable scavenging properties and ability to abrogate reactive oxygen species production via the p38-MAPK/JNK pathway, as well as increase the expression of endogenous nuclear factor erythroid 2-related factor 2 and heme oxygenase [199].

Nevertheless, it is important to underline that some polymeric coatings can induce allergic reactions and that the complete degradation of some polymeric coatings, such as poly(lactic-co-glycolic acid) in lactic and glycolic acids, takes months; therefore, their accumulation can lead to disturbances in the microenvironmental pH [199]. Given this knowledge, it is urgent to determine the polymer degradation rate and long-term safety of polymeric drug delivery systems intended for clinical use.

#### 4.1.2. Exosome Coatings

Milk exosomes are another promising strategy once they can take on a nanosize and can be easily customized depending on what they encapsulate [200]. Moreover, they are non-toxic, present a reduced immune response, and can confer protection on circulating genetic material; like other nanoencapsulation coatings, they feature remarkable biocompatibility and tumor targeting [201]. In addition, due to their high stability at lower pH values, milk exosomes can be used as carriers for oral drug delivery, enabling a wide range of preventive and therapeutic applications [174]. Recently, Barkallah et al. [200] demonstrated that exosomes loaded with 10 µg/mL delphinidin are non-toxic to human aortic endothelial cells, and can correct their nitric oxide levels and reduce angiogenesis, after 1 day of treatment. Moreover, Aqil et al. [201] have previously shown that exosomes in combination with anthocyanins at a concentration of 75 µM increase the potential of these phenolics to arrest the growth of A2780, A2780/CP70, OVCA432, and OVCA433 ovarian cancer cells, decrease the P-glycoprotein expression, and reduce the effective cisplatin concentration required to inhibit cisplatin-resistant ovarian cancer cells, after 3 days of treatment. In addition, exosomes composed of anthocyanins from blueberries also showed higher antiproliferative effects than the unencapsulated anthocyanins on colon cancer cell lines HCT-116 and HT-29 at concentrations between 25 and 200 µM, after 24 h of treatment [202], and on human lung cells A549 and H1299, breast cells MDA-MB-231 and MCF7, pancreatic cells PANC1 and Mia PaCa2, prostate cells PC3 and DU145, colon cells HCT-116, and ovarian cells OVCA432, at concentrations ranging from 20 to 100 µM, after 72 h of treatment [174].

However, it is important to take into account that exosomes present a limited transfection efficiency.

#### 4.1.3. Nanolipid Coatings

The nanoencapsulation of anthocyanins with lipids has also been extensively studied owing to their great biocompatibility, small size, high surface-to-volume ratio, and low fusibility [177]. In addition, their synthesis does not require the use of many solvents or aggregation with other nano delivery systems and can be manufactured on a large scale with high reproducibility and provide remarkable protection against enzymatic degradation, allowing the creation of a complex with unique physicochemical properties that can deliver the therapeutic molecule directly to target tissues or cells, minimizing its concentration and frequency of treatment [192]. The lipid chosen to coat the active molecules depends on many factors, such as the type of bioactive molecules, cosurfactants and surfactants, lipids, and cryoprotectants, as well as the target of the loaded bioactive molecule(s). Nowadays, many studies focus on the nasal delivery of drugs and phytochemicals, e.g., for the treatment of Alzheimer’s disease. As far as we know, nanoencapsulation of anthocyanins from elderberries, at a concentration of 0.5 mg/mL, with lipids showed a remarkable ability to modulate mitochondrial functionality, by enhancing complexes I and II of the mitochondrial respiratory chain and preserving the mitochondrial membrane potential in the presence of rotenone thanks to their ability to target mitochondria and protect these cells from rotenone and glutamate-induced toxicity [192].

Recently, lipid-derived nano-niosomes systems have also attracted considerable attention because they increase chemical stability and are economical and practical [5]. Anthocyanins encapsulated in niosomes, from black carrots, at concentrations of 6.25 and 100 µg/mL, showed the ability to be released after 10 h and decreased the viability of neuroblastoma cells Neuro 2A, after 2 days of treatment [203], while anthocyanins extracted from *Zea mays* and *Clitoria ternatea* (2–2000 µg/mL) showed a remarkable ability to increase collagen production in human gingival fibroblasts and permeate through the esophageal mucosa [163]. In addition, anthocyanin-added nano-niosomes (1%, 2%, 5%, and 10%, *v/v*) were reported to be non-toxic to normal HT22 hippocampal neuronal cells after 1 day of exposure, and fully internalized in BV2 microglial cells [204].

However, one of the major concerns of using lipids as nanocarriers is their cytotoxic potential due to their ability to create non-specific uptake [192].

#### 4.1.4. Nano-Polysaccharide Coatings

Certain types of polysaccharides are also used to encapsulate anthocyanins, as some of them have a charged residue and can therefore be easily linked to the opposite charge of the phospholipid residue, thus preventing phospholipid hydrolysis in the presence of enzymes and under acidic pH conditions [13,205]. Among them, chitosan and pectin are the most commonly used because both are non-toxic, environmentally friendly, biodegradable, and have high biocompatibility [28]. In addition to their potential advantages, they appear to be a promising, effective drug delivery system to the colon. In in vitro studies, nanoencapsulation of anthocyanins at concentrations of 5, 10, 20, and 40 µg/mL with pectin and chitosan showed greater potential to protect normal rat kidney (NRK) cells from acrylamide-induced damage than non-encapsulated anthocyanins and allowed their controlled release via the gastrointestinal tract [9]. They decrease reactive oxygen species, matrix metalloproteinases, and glutathione levels and provide protection to normal human hepatocyte L02 cells from palmitic acid-induced damage at concentrations ranging from 1.6 to 8.0 µM after 24 h of exposure [176]. Nevertheless, it is important to emphasize that chitosan dissolves only in certain dilute acidic solutions. Chondroitin sulfate is another promising alternative, as it is able to enhance the ability of black soybean anthocyanins at concentrations of 4, 7.5, and 15 µM to scavenge reactive oxygen species in human colon cells HCT-116, after 2 days of treatment [206] and to suppress human cervical cancer HeLa cells more effectively compared to anthocyanins alone, both at 100 µM after 1 day [20]. The combination of chitosan and chondroitin sulfate-loaded black rice anthocyanins at concentrations of 10 and 50 µg/mL also showed remarkable effects on human colon cancer cells HCT-116 by promoting negative changes in mitochondria and, consequently, apoptosis after four hours of exposure [207]. Finally, hyaluronic acid, a natural polysaccharide, together with 20 µg/mL cornflower anthocyanins, can enhance CD44^+^ apoptosis in colon cancer cells HT29, after 1 h of treatment [208].

Despite this, the employment of polysaccharide coatings shows some drawbacks, such as their cost, undesirable aggregation, and burst release promoted by their small particle size and large surface [209].

#### 4.1.5. Liposome Coatings

Nanoencapsulation of anthocyanins with liposomes has also been investigated, with most studies having shown that they can be used as nanocarriers for drug delivery due to their self-assembling and amphiphilic properties, flexibility, biocompatibility, biodegradability, longer duration of circulation, and low immunogenicity [183]. In addition, since they are composed of an aqueous core and a spherical hydrophobic shell, they can transport hydrophilic, hydrophobic, and amphiphilic molecules, as well as easily cross the blood–brain barrier [173]. Regarding their encapsulation with anthocyanins, Homayoonfal et al. [178] showed that they can enhance the antioxidant capacity of anthocyanins in a very remarkable and effective manner, as well as increase the metabolite activity and replication of human mesenchymal MSC and fibroblast FBL stem cells, at concentrations of 0.5 mg/mL and 10.4 µg/mL, respectively, after 1 week of treatment. Moreover, it has been also reported that there is a positive correlation between the size of nanoliposome particles and the encapsulation efficiency [161].

Nevertheless, it is important to note that liposomes can trigger undesirable immune responses, present a moderate loading capacity, and possibly crystallize after prolonged storage times [209].

#### 4.1.6. Halloysite Nanotubes Coatings

The use of halloysite nanotubes is also increasing worldwide, due to their unique properties that include particle size, high surface area, and surface-to-volume ratio, and very high dispersion rate [193]. Regarding anthocyanins, they have shown to be effective in suppressing the growth of human breast cancer MCF-7 and human colon cancer HT-29 at a concentration of 500 µg/mL compared to non-encapsulated anthocyanins, after one and 2 days of exposure [8]. Nonetheless, it is important to consider that halloysite nanotubes are inorganic and present toxicity and poor biocompatibility [193].

#### 4.1.7. Dendrimers Coatings

Dendrimers are another promising alternative, as they can reduce toxicity and improve water solubility, as well as possessing pharmacokinetic properties and biodistribution [209]. In addition, dendrimers can also be adjusted in size and shape, in order to favor surface area, loading, and delivery efficiency. They are usually composed of poly(amidoamine), poly(L-lysine), polyamides, polyethers, polyesters, poly(2,2-bis(hydroxyl methyl) propionic acid, and polypropylenimine. Together with anthocyanins, dendrimers can increase, dose-dependently, the cytotoxicity of neuro-2A brain neuroblastoma cell, after 24 h of treatment [194].

However, it is important to note that dendrimers present a complex synthetic route and can only carry low amounts of active molecules.

#### 4.1.8. Nanoemulsions Coatings

Nanoemulsions are another interesting drug delivery system, since their production is economic and they show high loading capacity and efficiency to carrier hydrophobic molecules, as well as a notable capacity to protect bioactive molecules against degradation. In addition, they can be easier manipulated to ameliorate drug release. Nonetheless, their use usually requires conjugation with nonionic surfactants to provide better stability [172]. Nonionic surfactants are recommended because they are safer than their ionic equivalents and are usually accepted for oral administration [172]. Recently, Nazareth et al. [165] reported that nanoemulsions of anthocyanins (400–1050 µg/mL) could be a promising alternative to conventional antibiotics. They demonstrated a remarkable ability to inhibit biofilm formation in Pseudomonas aeruginosa and Yersinia enterocolitica, and anti-quorum sensing activity against Chromobacterium violaceum, in a dose-dependent manner.

Nevertheless, it is important not to forget that some nanoemulsions present some non-compatibility, low viscosity, and can irritate skin.

### 4.2. In Vivo Studies

In vivo studies focusing on the employment of anthocyanins in nanocarriers for nano delivery are still scarce. Nonetheless, some up-to-date reports show promising data involving anthocyanins coated with nano-polymeric, dendrimers, carbohydrates, polysaccharides, and nano-niosomes with notable antioxidant, neurological and cardiovascular properties, and anticancer effects with no apparent adverse effects and, thus, with the potential to be marketed, as mentioned below.

#### 4.2.1. Nano-Polymeric Coatings

However, the use of polymer-based nanocarriers loaded with anthocyanins, at doses of 0.5 and 1 mg/g body weight, has already been reported. In these conditions, anthocyanins had shown the ability to protect fish models exposed to the pesticide cypermethrin from DNA degradation by increasing the penetrability of anthocyanins in liver, kidney, muscle, brain, and ovarian/testicular tissue, and decreasing cytotoxicity by modulating the antioxidant enzymes superoxide dismutase, catalase, and lipid peroxidase, after 7 days of treatment [197]. Moreover, complete tumor ablation was verified in MCF-7-bearing nude mice treated with anthocyanins at 250 µg/mL loaded into polymeric nanoparticles, at doses of 0.5 and 1 mg/g body weight, after 26 days of treatment [166].

#### 4.2.2. Dendrimers Coatings

Anthocyanins encapsulated in exosomes (5 mg anthocyanins and 50 mg exosome protein/kg body weight administered orally by gavage five times a week for a period of 3 weeks) also showed a remarkable ability to reduce tumor size in mice transplanted with A549 human lung cancer cells, with no apparent adverse effects [174]. In addition, they can also reduce the proliferation of colorectal cancer in Apc^Min/+^ mice inoculated with enterotoxigenic Bacteroides fragilis bacteria and treated for 4 weeks, 3 days per week, with a dose of 8.6 mg/kg/day, by increasing the activity phase II enzymes glutathione S-transferase Mu 1 and uridine 5′-diphospho-glucuronosyltransferase family 1 member A6 and decreasing the expression of aryl hydrocarbon receptor nuclear translocator 1, aryl hydrocarbon receptor, and cytochrome P450 family 1 subfamily A member 1 [202].

#### 4.2.3. Carbohydrate Coatings

Carbohydrate-based derivatives, especially chitosan, have been intensively studied, mainly due to their high loading efficiency, water solubility, and biocompatibility [29]. For example, Chatterjee et al. [175] reported that the use of anthocyanins loaded with chitosan (at a dose of 600 mg/kg body weight/day) from black carrot resulted in the remarkable ability to increase the activity of superoxide dismutase and catalase enzymes in rats. In addition, they can also enhance the potential of cyanidin 3-*O*-glucoside (5 mL/20 g body weight) to attenuate the oxidative stress induced by selenite sodium in rats treated four times daily for 1 week, enhance the transepithelial transport of liposomes to a depth of 40 mm in the cornea of rats, prolong the residence time on the cornea, and improve permeability in the corneal epithelium, thus increasing superoxide dismutase and catalase activity and decreasing glutathione activity and lipid peroxidation in the lens [205]. Moreover, they can also confer to this anthocyanin a higher ability to balance the B-cell lymphoma-2/leukemia-2 ratio and reduce UVB-induced epidermal damage, by decreasing lipid peroxidation, malondialdehyde, and 8-hydroxy-2′-deoxyguanosine and increasing visual and histological appearance and skin hydration, after 1 day of treatment [210]. In addition, Zhao et al. [9] reported that the use of chitosan combined with pectin can provide the ability of blueberry anthocyanins, at a concentration of 320 µg/mL, to increase the lifespan and reproductive capacity and improve the flexible locomotion behavior of *Caenorhabditis elegans* from damage induced by acrylamide, heat shock, oxidative stress, and ultraviolet light, and reduce ageing effects.

However, one of the major drawbacks of using carbohydrates as nanocarriers is related to their toxicity.

#### 4.2.4. Polysaccharide Coatings

On the other hand, the use of polysaccharides nanocarried with anthocyanins from black soybean for 5 days showed efficacy in reducing the tumor size of mice previously injected with human cancer cells HCT-116, increasing the number of apoptotic cells, and also contributing to the release of drugs at target sites [206]. In particular, cornflower anthocyanins loaded into the hyaluronic acid were found to be highly effective in increasing myelosuppression in mice transplanted with human colon cancer cells HT29 and treated for 13 days [208]. In addition, Hanafy [14] reported that encapsulation of these phytochemicals with bovine serum albumin can ameliorate cardiomyopathy, completely remove glycogen from tissues, and reduce malondialdehyde and collagen fibers in a fibrotic mouse model treated twice a week for 4 weeks.

#### 4.2.5. Nano-Niosomes Coatings

Nano-niosomes are another promising strategy, mainly thanks to their non-toxicity, greater stability, biocompatibility, capacity of penetration, and potential to control and target bioactive molecule(s) delivery [203]. So far, their encapsulation with anthocyanins has already shown remarkable healing activity in oral cavity wounds of rats [163]; the ability to improve insulin resistance and glucose intolerance, by increasing the anti-hyperglycemic/anti-diabetic ratios with no apparent toxicity; and lowering animal weight and plasma levels of insulin, glucose, leptin, and total cholesterol in obese mice at 300 mg/kg of body weight [4]. Moreover, nanoencapsulation of anthocyanins with nano-niosomes showed pronounced neuroprotection. For example, nano-niosomes can give anthocyanins a remarkable ability to cross the blood–brain barrier, reach it, and accumulate in mice with Alzheimer’s disease treated for 14 days, thus preventing tau hyperphosphorylation and reducing the expression of amyloid beta, beta-site amyloid precursor protein cleaving enzyme 1, and advanced glycation end products receptor, and neuroinflammatory and oxidative stress markers via the GSK-3β/CDK5 pathway [204]. Moreover, in the Aβ_1–42_ mouse model of Alzheimer’s disease, they may enhance the ability of anthocyanins (12 µg/g) to ameliorate memory impairment, as well as protect their pre- and postsynaptic proteins from Aβ_1–42_-induced synaptic dysfunction, regulate the p-PI3K/p-Akt/p-GSK3β pathway, prevent the hyperphosphorylation of tau protein at serines 413 and 404, and inhibit apoptosis during 14 days of treatment [211]. Although they seem to be a hopeful strategy, it is still difficult to find an appropriate concentration able to balance the relationship between the entrapment efficiency and the affinity of the cell membrane.

However, the development of nano-niosomes is expensive and there exists the risk of aggregation; in addition, nano-niosomes present low loading capacity [163].

**Table 2:** In vitro and in vivo studies on the potential therapeutic effects of drug delivery systems nanocarried with anthocyanins.

Source	Nanoencapsulation Method	Nanoencapsulating Agent(s)	Main Achievement	Reference
In vitro studies				
Polymeric-based				
Pelargonidin	Standard solvent displacement technique	Poly(lactic-co-glycolic acid)	■ improved glucose homeostasis signaling cascades by ↓ GLUT4, IRS1, IRS2, PI3, GK, and PK protein expression in alloxan-induced hyperglycemic skeletal muscle L6 cells ■ initiated cytochrome complex-mediated apoptosis in alloxan-induced hyperglycemic skeletal muscle L6 cells	[196]
Pelargonidin	Displacement technique	Poly(lactic-co-glycolic acid)	↑ L6 muscle cells viability exposed to cypermethrin pesticide, by inhibiting ROS generation ■ protected these cells against nuclear damage	[197]
Pelargonidin	Solvent displacement technique	Poly(lactic-co-glycolic acid)	■ prevented alloxan-induced DNA damage in L6 cells via activation of PARP and p53 and protected them against oxidative stress	[198]
Not mentioned	Emulsification-solvent evaporation technique	Poly(lactic-co-glycolic acid)	*■* no cytotoxic effects on human neuroblastoma SH-SY5Y cells ↑ SH-SY5Y cells’ viability against A*β*_1–42_ due to their markedly free radical scavenging properties and abrogated reactive oxygen species generation via the p38-MAPK/JNK pathways ↑ induced Nrf2 and HO-1 expression ↓ Alzheimer’s markers APP, BACE-1, neuroinflammatory markers *ρ*-NF-kB, TNF-*α* and iNOS, and neuroapoptotic markers Bcl2, Bax, and caspase-3 protein expressions	[199]
Exosome-based				
Delphinidin	Extracellular vesicles	Small extracellular vesicles from JAWS II cells	■ no cytotoxicity effects on human aortic endothelial cells ■ corrected the levels of nitric oxide in these cells ↓ angiogenesis in these cells	[200]
Anthocyanins from bilberries	Dissolution and mixing methods	Exosomes isolated from mature bovine milk	↓ A2780A2780/CP70, OVCA432 and OVCA433 ovarian cancer cells’ growth ↓ P-glycoprotein expression ↓ the dose of cisplatin required to stop the mentioned ovarian cancer cells’ growth	[201]
Anthocyanins from bilberries	Dissolution and mixing methods	Exosomes isolated from mature bovine milk	↑ anti-proliferative and anti-inflammatory effects on human lung A549 and H1299, breast MDA-MB-231 and MCF7, pancreatic PANC1 and Mia PaCa2, prostate PC3 and DU145, colon HCT-116 and ovarian OVCA432 cancer cells	[174]
Anthocyanins from bilberries	Dissolution and mixing methods	Exosomes isolated from mature bovine milk	↑ antiproliferative effects on colon HCT-116 and HT-29 cancer cell lines ■ no cytotoxicity effects on normal CCD-18Co colon cells	[202]
Polysaccharide-based				
Pelargonidin 3-*O*-glucoside	Thin-film hydration method combined with probe sonication	Chitosan and pectin	↓ palmitic acid-induced hepatocytes injury in normal human hepatocytes L02 cells by suppressing reactive oxygen production, superoxide generation, MMP collapse ↓ GSH levels	[176]
Anthocyanins from bilberries	Complexation	Chitosan and pectin	↑ storage stability under light and dark conditions *■* improved potential to escape from the interference of stomach acid environment and released slowly in small intestinal fluid and, hence, can be delivered in a controlled way along with the gastrointestinal tract *■* protected anthocyanins from degradation by gastric acid *■* protected normal rat kidney NRK cells against acrylamide-induced damage	[9]
Anthocyanins from black soybean	Complexation	Chondroitin sulfate	■ notable potential to scavenge reactive oxygen species in human colon HCT-116 cells	[206]
Anthocyanins from black soybean	Complexation	Chondroitin sulfate	↑ structural stability ■ protected anthocyanins from degradation induced by hydroxyl molecules under pH > 9 ■ suppressed human cervical HeLa cancer cells in a more effective way when compared to anthocyanins alone	[20]
Anthocyanins from black rice	Ionic gelation method	Chitosan and chondroitin sulfate	■ negative changes in mitochondria, by ↑ apoptosis in human colon HCT-116 cancer cells	[207]
Lipid-based				
Anthocyanins from elderberries	Homogenization, evaporation, sonication, and hand-shaking methods	Lipids from *Codium tomentosum*	■ modulated mitochondria functionality in neuroblastoma SH-SY5Y cells, by ↑ mitochondrial respiratory chain complexes I and II, and preserved the mitochondrial membrane potential in the presence of rotenone thanks to their ability to reach mitochondria ■ protected neuroblastoma SH-SY5Y cells against rotenone and glutamate-induced toxicity	[192]
Nano-niosome gel				
Anthocyanins from *Zea mays* and *Clitoria ternatea*	Thin-film method and hand-shaking methods	Sodium polyacrylate and carbomer 934P (G.M.P.), cholesterol, Span60, fluocinolone acetonide	↑ collagen production in human gingival fibroblasts ↑ permeation through esophageal mucosa	[163]
Liposome-based				
Anthocyanins from barberries	Combination between hydration and ultrasound processes	Rapeseed lecithin	■ no cytotoxic effects on human mesenchymal MSC and fibroblast FBL stem cells ↑ metabolite activity, cells’ replication, and antioxidant capacity	[178]
Halloysite nanotubes-based				
Anthocyanins from black carrot	Vacuum	Alumina–silicate sheets	↓ in 2-fold the growth of human breast cancer MCF-7 and human colon cancer HT-29 when compared to non-encapsulated anthocyanins ■ control of anthocyanins’ release can be achieved by pH	[8]
Dendrimer nanoparticles-based				
Anthocyanins from black carrot	Sol-gel technique	Silica–PAMAM dendrimer	↓ cell survival of neuro 2A brain neuroblastoma cells	[194]
Nanoemulsions-based				
Anthocyanins from *Carissa spinarum*	Complexation	Glycerol–sodium benzoate and Tween 80 as surfactant	■ anti-quorum sensing activity against *Chromobacterium violaceum* ■ inhibited biofilm formation in *Pseudomonas aeruginosa* and *Yersinia enterocolitica*	[165]
Anthocyanins from black carrot	Double emulsion method	Polycaprolactone	■ anthocyanins’ release after 10 h ↓ neuroblastoma Neuro 2A cells viability	[203]
Not mentioned	Homogenization, stirring and centrifugation methods	Poly(ethylene glycol)-gold nanoparticles	■ no cytotoxicity effects on normal HT22 hippocampal neuronal cells ■ complete internalization in BV2 microglial cells	[204]
Hyaluronic acid-based				
Anthocyanins from cornflower	Homogenization	Sulfuric hyaluronic acid	■ improved apoptosis of CD44^+^ in colon cancer HT29 cells	[208]
In vivo studies				
Polymeric-based				
Pelargonidin	Displacement technique	Poly-lactide-co-glycolide acid	↑ penetrability through liver, kidney, muscle, brain, and ovary/testis tissue of fish models exposed to cypermethrin pesticide ↓ cytotoxicity in them, by modulating antioxidative enzymes superoxide dismutase, catalase and lipid peroxidase and, hence, protecting fish models against cypermethrin pesticide damage and protecting them against DNA depletion	[197]
Anthocyanins from honeysuckle fruits	One-pot method	Fe^III^ ions and poly(L-glutamic acid)-g-methoxy poly(ethylene glycol)	↑ permeability, retention, and renal clearance ■ complete tumor ablation in MCF-7-bearing nude mice	[166]
Exosomes				
Anthocyanins from bilberries	Dissolution and mixing methods	Exosomes isolated from mature bovine milk	■ significant growth inhibition regarding tumor size in mice that were transplanted with human lung cancer A549 cells, without any apparent undesirable side effects	[174]
Anthocyanins from bilberries	Dissolution and mixing methods	Exosomes isolated from mature bovine milk	↓ colorectal cancer proliferation in Apc^Min/+^ mice inoculated with *Enterotoxigenic Bacteriodes fragilis* bacteria ↓ ARNT1, AhR, and CYP1A1 expression in mice ↑ phase II enzymes GSTM1 and UGT1A6	[202]
Carbohydrate-based				
Cyanidin 3-*O*-glycoside	Reverse-phase evaporation method	Chitosan	■ attenuate oxidative stress induced by selenite sodium in rats ■ improved transepithelial transport of liposomes to a depth of 40 mm in rat’s cornea, prolonged residence time on the cornea, and ↑ permeability in the corneal epithelium ↑ superoxide dismutase and catalase activity and ↓ glutathione activity and lipid peroxidation in rat’s lens	[205]
Cyanidin 3-*O*-glucoside	Ionic gelation	Chitosan and sodium tripolyphosphate	■ slower release at higher pH ■ balanced the B-cell lymphoma-2/leukemia-2 ratio ↓ UVB-induced epidermal damage through the p53-mediated apoptosis signaling pathway, by ↓ UVB-induced lipid peroxidation, malondialdehyde, and 8-hydroxy-2′-deoxyguanosine contents, and downregulate p53, Bax, and caspases- 3 and 9 expression ↑ the visual and histologic appearance, skin moisture, and apoptotic index under UVB exposure	[210]
Anthocyanins from bilberries	Complexation	Chitosan and pectin	■ protected *Caenorhabditis elegans* from damage induced by acrylamide, heat shock, oxidative stress, and ultraviolet light ■ complexation ↑ lifespan, reproductive capability, and offers a flexible locomotion behavior in this animal model■ complexation ↓ autofluorescent lipofuscin particles and exhibits anti-aging effects in this animal model	[9]
Anthocyanins from black carrot	Ionic gelation technique	Chitosan	↑ superoxide dismutase and catalase enzyme activities in rats	[175]
Polysaccharide-based				
Anthocyanins from black soybean	Complexation	Chondroitin sulfate	■ tumor size of mice that were injected with human cancer HCT-116 cells did not change after 5 days of treatment ↑ apoptotic cells ■ tumor targeted and drug released	[206]
Hyaluronic acid-based				
Anthocyanins from cornflower	Homogenization	Sulfuric hyaluronic acid	↓ myelosuppression in mice that were transplanted with colon cancer HT29 cells	[208]
Lipid hybrid-based				
Anthocyanins from red beets	Homogenization	Bovine serum albumin	■ ameliorated cardiomyopathy in fibrotic mice model ■ eliminated glycogen completely from tissue ↓ malondialdehyde and collagen fibers	[14]
Nano-niosome based				
Anthocyanins from *Zea mays* and *Clitoria ternatea*	Thin-film method and hand-shaking methods	Sodium polyacrylate and carbomer 934P (G.M.P.), cholesterol, Span60, fluocinolone acetonide	■ healing activity onto incisional wounds in oral cavities of rats	[163]
Anthocyanins from *Vaccinium Meridionale*	Commercial pronanosome precursor (Nio-N de NANOVEX Biotechnologies S.L, Asturias, España), involving mixture and incubation techniques, and a dispersion using an ultrasound probe	Niosomes	■ ameliorated insulin resistance and glucose intolerance in obese mice ↓ animal weight and plasma insulin, glucose, leptin, and total cholesterol levels in them ↑ anti-hyperglycemic/anti-diabetic ratio, without any apparent toxicity	[4]
Not mentioned	Homogenization, stirring, and centrifugation methods	Poly (ethylene glycol)-gold nanoparticles	■ coated anthocyanins can successfully cross the blood–brain barrier, reach, and be accumulated in Alzheimer’s disease mice model ■ prevent tau hyperphosphorylation and ↓ Amyloid beta, BACE-1, and RAGE expressions, and reduce neuro-inflammatory and oxidative stress markers via GSK-3β/CDK5 pathway	[204]
Antocyanins from from black beans	Dissolution and stirring methods	Polyethylene glycol-with gold nanoparticles	■ ameliorated memory impairments in the Aβ_1–42_ mice model of Alzheimer’s disease in a more efficient way than free-anthocyanins ■ protected pre- and postsynaptic proteins from Aβ_1–42_-induced synaptic dysfunction in Aβ_1–42_-injected mice ■ regulated the p-PI3K/p-Akt/p-GSK3β pathway, preventing the hyperphosphorylation of tau protein at serines 413 and 404 in the Aβ_1–42_- mice model of Alzheimer’s disease ■ inhibited apoptosis in the Aβ_1–42_- mice model of Alzheimer’s disease	[211]

↑: increase; ↓: decrease; Nrf2: endogenous nuclear factor erythroid 2-related factor 2; HO-1: heme oxygenase 1; BACE-1: beta-site amyloid precursor protein cleaving enzyme 1; RAGE: receptor for advanced glycation end products; APP: amyloid precursor protein; *ρ*-NF-kB: phospho-nuclear factor kappa B; TNF-*α*: tumor necrosis factor; iNOS: inducible nitric oxide synthase; Bcl2: B-cell lymphoma 2; Bax: bcl-2 associated X-protein; ARNT1: aryl hydrocarbon receptor nuclear translocator 1; AhR: aryl hydrocarbon receptor; CYP1A1: cytochrome P450 family 1 subfamily A member 1; GSTM1: glutathione S-transferase mu 1; UGT1A6: uridine 5′-diphospho-glucuronosyltransferase family 1 member A6, GLUT4: glucose transporter type 4; IRS: insulin receptor substrate; PI3: peptidase inhibitor 3; GK: glycerol kinase; PK: protein kinase; MMP: matrix metalloproteinases; GSH: glutathione; PARP: poly(ADP-ribose) polymerase; PAMAM: poly(amidoamine).

## 5. Conclusions

Given the worldwide increase in chronic diseases and the urgent need to search for new, effective, safe, and low-toxicity drugs, it is not surprising that many phytochemicals, such as anthocyanins, are considered good candidates. In fact, their pyrogallol, methoxy, and catechol groups confer remarkable health-promoting properties to anthocyanins. Therefore, and considering their limitations (e.g., high sensitivity to pH, rapid absorption, and metabolization), encapsulation seems to be a promising approach. In addition, the combination with other bioactive molecules/pharmaceuticals also appears to be a promising strategy in order to improve the therapeutic effects. Nevertheless, it is imperative to know their safe dosage in humans, since they may act as pro-oxidants under certain conditions, and because in many situations the use of higher dosages may not help to increase their biological potential. Furthermore, it is also important to note that nanoparticles manufacturing needs to be more scalable and robust, and performed under stringent, specific guidelines. Furthermore, clinical studies need to be accurately designed, detailed measured, and controlled. Finally, statistical analyses need to be adequately applied.

## Data Availability

Not applicable.
